# The collaborative development of graduate education and high economic quality and its dynamic evolution forecast

**DOI:** 10.1016/j.heliyon.2023.e21438

**Published:** 2023-10-30

**Authors:** Huang Zhiqi, Sun Fan, Zhou Yangmei, Li Yan, Li Zheng

**Affiliations:** a School of Management and Economics, North China University of Water Resources and Electric Power, Zhengzhou 450046, China; b School of Business Administration, Henan University of Economics and Law, Zhengzhou 450046, China; c Faculty of Business and Economics, University of Melbourne, Parkville 3010, Victoria, Australia

**Keywords:** Graduate education, High-quality economic development, Coordinated development, Dynamic evolution prediction

## Abstract

To promote the coordinated development of high quality postgraduate education and economy is the realization of all-round construction of the socialist modernization of China power.Based on the panel data of 30 provinces in China from 2009 to 2021, this paper constructs the index of graduate education and high-quality economic development respectively, and analyzes it by coupling coordination model, kernel density estimation and spatial Markov chain model.The results show that: first, the postgraduate education and the high economic quality of most provinces in China are matched, but there is a significant difference between the eastern and western regions in their collaborative development; Second, the level of synergistic development between graduate education and high economic quality is low, and the central and northeastern regions are seriously divided, but the differences in synergistic effects between different regions are narrowing.Third, according to the results of trend prediction, there is the coexistence of "beggar-thy-neighbor" and "good-neighborliness", and it is difficult to achieve leapfrog coordinated development.Therefore, it is proposed to pay attention to the high-quality economic development to provide a rich material basis for graduate education, the change of graduate education structure should adapt to the needs of high-quality economic development, and implement different reform measures in different regions to promote the comprehensive construction of a modern socialist country.

## 1 Introduction

In the 20th National Congress of the Communist Party of China held in October 2022, it was clearly stated that education is the basic support for comprehensively building a modern socialist country, and education is placed in a very high strategic position [1], and graduate education is at the top of China's education, bearing the important mission of providing high-end talents and scientific and technological innovation [2]. In 2010, 492,800 graduate students were enrolled in China, while in 2022, the number of graduate students in China will reach 1.242,500 million, an increase of 5.61 % over 2021 [3]. This is a good start in the period of the 14th Five-Year Plan for China's national economic and social development.

At the same time, another key task in the period of China's 14th Five-Year Plan is high-quality economic development [4]. In 2009, China's per capita GDP reached 25,545 yuan, and in 2022, China's per capita GDP reached 85,698 yuan, an increase of 3 % over 2021 [5], and China's economic development has achieved remarkable results. However, compared with other developed countries in the world, China's per capita GDP in 2022 ranks only 63rd in the world [6], and China's development still needs to strive forward. Among them, how to promote the coordinated development of graduate education and economic quality is the key to realize the comprehensive construction of a modern socialist country. China needs to develop both education and economy in the next step. The two go hand in hand.

However, few existing literatures pay attention to the synergic development of graduate education and high economic quality. In order to study the current situation of graduate education and high economic quality in China, and hope to provide empirical evidence and policy suggestions for the synergic development of graduate education and high economic quality in China and even the world, this paper will sort out and summarize the relevant researches of existing literatures. The panel data of 30 provinces in China from 2009 to 2021 are taken as an example to conduct coupling coordination analysis and dynamic evolution prediction.

## 2 Literature review

### 2.1 Research on graduate education

In 2012, the 18th Congress of the Communist Party of China pointed out that China has entered the ranks of the world's major countries in postgraduate education [7], which means that although China's postgraduate education started relatively late compared with European and American countries [8], it has a strong momentum of development. Referring to the existing literature, some scholars have standardized max-min data processing from the five aspects of input, output, structure, internationalization and satisfaction, and then measured by entropy weight method to find that China's graduate education was in a good state in 2017 [9]. Through the analysis of the number of graduate education institutions and Arc GIS software, some scholars also found that there are significant regional differences in China's graduate education: the graduate education level in coastal and high-latitude areas is higher [10]. Considering that the larger the scale of postgraduate education [11], the more complete the education structure [12], the higher the quality of education [13] and the better the educational benefits [14], the higher the level of postgraduate education, this paper measures the postgraduate education development index and analyzes it by geographical region.

### 2.2 Related research on high-quality economic development

Since high-quality economic development was first proposed in the report of the 19th National Congress of the Communist Party of China in 2017, the research on high-quality economic development has been relatively complete. In the existing literature, some scholars have measured the level of high-quality economic development in various regions of China from five aspects: supply, demand, efficiency, operation and opening-up. Studies have shown that there are significant differences between eastern and western China [15]. Some scholars also start from the five aspects of innovation, coordination, green, openness and sharing, and their research shows that in terms of provincial panel data, China's high-quality economic development is on the rise and the inter-regional gap is significantly narrowing [16]. Some scholars, starting from input-output indicators and using data envelopment analysis (DEA), show that the high-quality development level of provincial economy in China is relatively stable and has a large room for improvement [17]. Based on the new development concept that is more in line with the development background of The Times, this paper also constructs the high-quality economic development index from the five development dimensions of innovation, coordination, green, open and sharing, and analyzes it by geographical region.

### 2.3 Research on postgraduate education promoting high-quality economic development

Both graduate education and high-quality economic development have great potential, and the internal mechanism of how to realize the synergistic development of the two is analyzed. In the existing literature, the theoretical logic of graduate education promoting high-quality economic development starts from five aspects. First, the "talent increment" and "resource stock" of graduate education will jointly promote high-tech innovation, and this promotion effect will be different in different provinces, resulting in differences in high-quality economic development [18]. Second, the structure of graduate education will promote the coordinated development of industrial structure, and the overall level is low, and the overall trend is upward, thus driving the high-quality development of social economy [19]. Thirdly, ecological civilization education for graduate students can promote the green development of agriculture and industry on a large scale, so as to realize the green development of social economy [20]. Fourth, postgraduate education, especially doctoral students, can better learn advanced western knowledge and technology, and then introduce it into China to promote high-quality economic development [21]. Fifth, graduate education can break the development imbalance between the eastern and western regions of China and make the exchange of talents more conducive to high-quality economic development [22]. Therefore, graduate education can effectively promote high-quality economic development from the five aspects of innovation, coordination, green, open and sharing.

### 2.4 Research on promoting graduate education with high quality economic development

The theoretical logic of promoting graduate education with high-quality economic development starts from scale, structure, quality and benefit. First, economic growth increases the scale of financial investment in graduate education, and there is a significant difference in financial investment between the eastern and western regions [23]. Secondly, the high-quality economic development determines the structure of postgraduate education, especially the increasing demand for the training of doctoral students in science and engineering [24]. Moreover, high-quality economic development requires the support of high-level talents, and the faster the economic development, the higher the quality of postgraduate education required [25]. Finally, high-quality economic development provides high-paying positions for high-level talents in society, which require corresponding abilities to match [26]. Therefore, high-quality economic development also effectively promotes graduate education.

### 2.5 Summary

To sum up, the existing literature is rich in research on graduate education, high-quality economic development and the one-way relationship between them, but few papers discuss the synergistic development effect of graduate education and high-quality economic development and its dynamic prediction analysis. Based on this, the marginal contribution of this paper is as follows: First, in terms of research content, this paper makes up for the deficiency of only analyzing the one-way effect between graduate education and high-quality economic development, and enriches the connotation of collaborative development; Second, in terms of research methods, this paper uses kernel density estimation and spatial Markov chain model to show the change of the synergistic effect over time, and also does trend prediction analysis. Third, in terms of research results, the analysis of graduate education and high-quality coordinated development of economy not only starts from regional differences, but also analyzes the differences of time.

## 3 Research design

In order to intuitively demonstrate the synergy between graduate education and high quality economy in appellate theoretical analysis, this chapter lays a foundation for the following empirical analysis through the selection of research methods, the construction of relevant indicators and data collection.

### 3.1 Research method

#### 3.1.1 Coupling coordination model

In order to investigate the synergistic effect between graduate education and high-quality economic development, this paper constructs coupling coordination degree through coupling coordination model with reference to Ruichao Nie et al. (2022) [27,28]. The specific model is shown as follows:

(1) $$C=\sqrt{\frac{A_{1}\times A_{2}}{{\left(\frac{A_{1}+A_{2}}{2}\right)}^{2}}}$$

(2) $$D=\sqrt{C\left(\alpha A_{1}+\beta A_{2}\right)}$$

In the above equations (1), (2), *A*_*1*_ stands for graduate education development index, *A*_*2*_ stands for high-quality economic development index; *C* represents the coupling degree, and the value is between [0.0 and 1.0]. The closer the value is to 1, the better the match between graduate education and high-quality economic development. *α* and *β* respectively represent the weight of graduate education and high-quality economic development in the whole system. Considering that synergies are mutual, both *α* and *β* are set at 0.5 in this paper. *D* represents the degree of coupling coordination, and the value is also between [0.0 and 1.0]. The closer the value is to 1, the closer the synergy between graduate education and high-quality economic development is. In order to show the degree of coupling coordination intuitively, this paper constructs the following Table 1 to divide 10 coordination levels.

**Table 1:** Coupling coordination degree classification.

Coordination level	Coupling coordination degree	Degree of coupling coordination
1	[0.0，0.1）	Hyperdysregulation
2	[0.1，0.2）	Severe disorder
3	[0.2，0.3）	Moderate dysregulation
4	[0.3，0.4）	Mild disorder
5	[0.4，0.5）	Borderline disorder
6	[0.5，0.6）	Forced coordination
7	[0.6，0.7）	Primary coordination
8	[0.7，0.8）	Intermediate coordination
9	[0.8，0.9）	Good coordination
10	[0.9，1.0]	Quality coordination

#### 3.1.2 Kernel density estimation

In order to better understand the time distribution characteristics of the synergistic effect between provincial graduate education and high-quality economic development in China, this paper adopts an important non-parametric estimation method, kernel density estimation, which reflects the distribution characteristics of coupling coordination degree through continuous and dense curves, and the graph of the vertical section of the time axis can reflect the level of coupling coordination degree in a year. The height and width of the wave crest of the curve can reflect the aggregation degree of the coupling coordination degree in a year, the number of wave peaks can reflect the polarization degree of the coupling coordination degree data, and the distribution ductility can reflect the gap between a certain region and other regions with the highest or lowest coupling coordination degree. The more serious the trailing, the greater the difference between regions. The parallel section with the time axis can reflect the dynamic change process of the same area in different time. The specific nuclear density curve is generated by equation (3) [29,30]:

(3) $$f_{m}\left(y\right)=\frac{1}{a_{m}N}\sum_{i=1}^{a_{m}}K\left(\frac{y_{mi}-y}{N}\right)$$

Where *a*_*m*_ represents the number of provinces in the desired region; *N* represents the number of samples, that is, the window width of the kernel density function. The smaller the value, the more accurate the function result is. *K*（.）Represents the kernel density function; *y*_*mi*_ represents the coupling coordination degree of *i* province in m region. y represents the mean of the coupling coordination degree.

#### 3.1.3 Spatial Markov chain prediction model

In order to predict the synergistic effect between provincial graduate education and high-quality economic development in China, this paper introduces the adjacency weight matrix in the spatial Markov chain to calculate the spatial lag value, so as to show the relationship between spatial units. The spatial Markov chain transition probability matrix can be divided into m m × m conditional transition matrices, and *p*_*ij*_ (m) represents the spatial transition probability value that transforms into type *j* at time t+1 based on the spatial lag type m of the spatial unit at time t. The spatial lag type of a spatial unit is determined by its spatial lag value, which is the spatially weighted average value of the neighbor area attribute value of the spatial unit, as shown in equation (4) [31,32]:

(4) $$Lag=Y_{i}W_{ij}$$

In the above formula (10), *Y*_*i*_ is the attribute value of the space unit; *W*_*ij*_ is the element in row *i* and column *j* of the spatial weight matrix *W*, that is, the relationship matrix between the space unit and the neighbor region.

### 3.2 Index construction

#### 3.2.1 High-quality economic development

The explained variable selected in this paper is the Comprehensive Index of high-quality economic Development (*HED*). With reference to the existing literature of Sun Hao et al. (2020) [33], the five development concepts are as follows: Based on "innovative development, coordinated development, green development, open development and shared development", each positive and negative index is indexed by the following equation (5) and equation (6), and then the information entropy of the five components is calculated by the proportion of each index and summed as shown in the following equation (7). Finally, the overall score is calculated by the total contribution of each index and the weight of each index fifth, as shown in equation (8):

(5) $$X_{ab}=\frac{x_{ab}-{x_{ab}}_{\min}}{{x_{ab}}_{\max}-{x_{ab}}_{\min}}$$

(6) $$X_{ab}=\frac{x_{ab_{\max}}-x_{ab}}{{x_{ab}}_{\max}-{x_{ab}}_{\min}}$$

(7) $$Y_{bc}=\sum_{a=1}(X_{ab}\times \frac{X_{ab}}{{sumX}_{ab}})$$

(8) $$W_{b}=\sum_{a=1}^{a=5}\left(\frac{1}{5}\times Y_{bc}\right)$$

In the above equation, *x*_*ab*_ represents the data of province *b* under index *a*;*x*_*abmax*_ and *x*_*abmin*_ represent the maximum and minimum values of *x*_*ab*_ data; *X*_*ab*_ represents the exponential data of province *b* under index *a*; *Y*_*bc*_ represents the sum of information entropy of different development concepts *c* of province *b*. *W*_*b*_ represents the comprehensive index of high-quality economic development of Province *b*, and the specific index composition is shown in Table A1.

#### 3.2.2 Graduate education

Regarding the construction of graduate education development index, this paper refers to Li Ping et al. [10]. (2022) and other practices from four dimensions of scale, structure, quality and efficiency, and its specific subdivision is shown in Table 2 above. Then, the data in this paper are standardized as shown in equation (5) above, and the entropy weight method is used to evaluate the graduate education development index from 2009 to 2021. The higher the value of the eight three-level indicators, the higher the graduate education development index.

**Table 2:** The result of coupling evaluation between graduate education and high quality economic development.

District		2009			2015			2021	
	Coupling degree	Coupling coordination degree	Evaluate	Coupling degree	Coupling coordination degree	Evaluate	Coupling degree	Coupling coordination degree	Evaluate
Beijing	0.8785	0.7322	8	0.9801	0.8776	9	0.9992	0.8782	9
Tianjing	0.6870	0.4272	5	0.7541	0.5100	6	0.8103	0.5423	6
Hebei	0.9470	0.2955	3	0.9937	0.3539	4	0.9823	0.4425	5
Shanxi	0.9464	0.2876	3	0.8587	0.3614	4	0.9335	0.3795	4
Neimenggu	0.8495	0.2596	3	0.9989	0.2976	3	0.8710	0.3900	4
Liaoning	0.9472	0.4050	5	0.9500	0.5218	6	0.9498	0.5342	6
Jilin	0.9997	0.2875	3	0.9995	0.3370	4	0.9804	0.4049	5
Heilongjinag	0.9585	0.3509	4	0.9995	0.3852	4	0.9647	0.4269	5
Shanghai	0.8097	0.6030	7	0.9322	0.6893	7	0.8776	0.6528	7
Jiangsu	0.9676	0.4955	5	1.0000	0.5955	6	0.9965	0.6581	7
Zhejiang	0.8892	0.4142	5	0.9739	0.5066	6	0.9639	0.5368	6
Anhui	0.9786	0.2715	3	0.9708	0.3987	4	0.9744	0.4756	5
Fujian	0.7933	0.3669	4	0.9245	0.4411	5	0.9749	0.4537	5
Jiangxi	0.9882	0.2384	3	0.9875	0.3343	4	0.9676	0.4181	5
Shandong	0.9550	0.3955	4	0.9987	0.4655	5	0.9945	0.5655	6
Henan	0.9942	0.2942	3	0.9979	0.3684	4	0.9994	0.4618	5
Hubei	0.9953	0.4105	5	0.9979	0.5246	6	0.9979	0.5110	6
Hunan	0.9780	0.3208	4	0.9999	0.3876	4	0.9977	0.4697	5
Guangdong	0.8777	0.4908	5	0.9688	0.5781	6	0.9913	0.6677	7
Guangxi	0.9665	0.2483	3	0.9856	0.3472	4	0.9305	0.4314	5
Hainan	0.3974	0.2686	3	0.6723	0.2782	3	0.7160	0.3665	4
Chongqing	0.9178	0.3153	4	0.9367	0.4108	5	0.9579	0.4609	5
Sicuan	0.9996	0.3347	4	0.9996	0.4304	5	0.9946	0.5396	6
Guizhou	0.9964	0.1661	2	0.9492	0.3014	4	0.9641	0.3701	4
Yunnan	0.9887	0.2615	3	0.9857	0.3505	4	0.9984	0.3768	4
Shanxi	0.9995	0.3218	4	0.9917	0.4491	5	0.9996	0.5017	6
Gansu	0.9040	0.2692	3	0.9450	0.3395	4	0.9457	0.3719	4
Qinghai	0.4987	0.1935	2	0.6814	0.2125	3	0.8225	0.2397	3
Ningxia	0.8296	0.1924	2	0.8597	0.1996	2	0.6496	0.2730	3
Xinjang	0.9994	0.1857	2	0.9993	0.2799	3	0.9943	0.2931	3

### 3.3 Data source

In this paper, the panel data of 30 provinces in China (excluding Hong Kong, Macao and Taiwan) from 2009 to 2021 are selected as samples, and the eastern, central, northeastern and western regions are divided according to national economic development. The specific data comes from China Statistical Yearbook, China Science and Technology Statistical Yearbook, China Energy Statistical Yearbook and China Education Statistical Yearbook. For some missing data values, this paper uses linear interpolation to supplement them.

## 4 Empirical analysis

### 4.1 The coupling and coordination analysis of graduate education development and high-quality economic development

#### 4.1.1 The change in the two indices at the national level

**Fig. 1:**
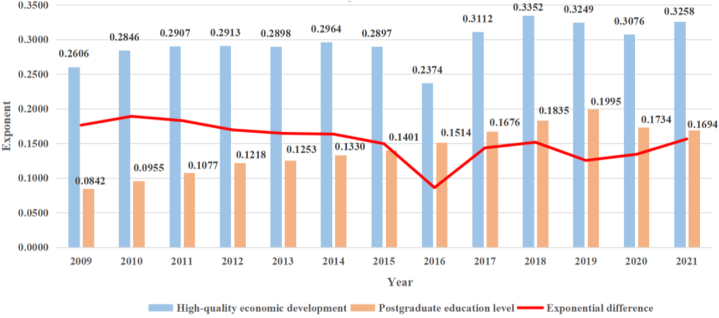
A comparison of graduate education and high-quality economic development across the country.

In order to visually show the changes between the graduate education Development Index and the economic quality Development Index, we averaged the indices of each province by year, as shown in Fig. 1 below.

In Fig. 1, from 2009 to 2015, both the level of high-quality economic development and the level of graduate education showed an overall upward trend, and the gap between the two was decreasing, thanks to the relevant support measures for education and economy in the 11th Five-Year Plan and the 12th Five-Year Plan. In 2016, challenges such as "artificial intelligence + education", the passage of the amendment to the Law on the Promotion of Private Education, and the plunge in the share price of the training institution giant "New Oriental" led to a decline in the level of postgraduate education. At the same time, problems such as Brexit and purchase and loan restrictions in China's real estate market led to a decline in the high-quality development level of China's economy, followed by a decline in the gap between the two. But this is not what we would expect from a reduction in the gap; The COVID-19 epidemic that swept the world at the end of 2019 has reduced the level of postgraduate education while lagging economic development, and has had a serious impact on the employment situation and international exchanges of postgraduate education, thus increasing the gap between the development level of postgraduate education and high-quality economic development after 2019.

#### 4.1.2 The result of coupling evaluation between graduate education and high quality economic development

In this paper, through the coupling coordination model, postgraduate education and high-quality economic development in 30 provinces in China from 2009 to 2021 are coupled, and 2009, 2015 and 2021 are selected as shown in Table 2 below.

In Table 2 above, it can be found that the coupling degree C between graduate education and high-quality economic development in most provinces is above 0.8, and even above 0.95 in most provinces, which means that graduate education and high-quality economic development are matched, and the result of coupling coordination degree obtained is reasonable. However, there are also some provinces, such as Hainan, which is always below 0.8, and the dependence between postgraduate education and high-quality economic development is relatively low. This may be due to the late start and weak foundation of education in Hainan Province, while the economy is significantly influenced by tourism, resulting in a weak dependence between the two. The coupling degree of other provinces has changed from less than 0.8 to higher than 0.8 over time, such as Tianjin, Fujian and Qinghai provinces, among which the coupling degree of Fujian in the eastern region has changed the most, which is inseparable from the eastern region's emphasis on the coordinated development of education and economy.

Secondly, the coupling coordination degree of Beijing, Shanghai, Guangdong and Zhejiang provinces in the eastern region ranked top in each year, which means that the postgraduate education in these four regions is closely related to the coordinated development of high quality economy. This is in line with the Several Opinions on Coordinating the Reform and Development of Higher Education in Beijing promulgated by Beijing Municipality, the Outline of Shanghai Medium and Long-Term Education Reform and Development Plan (2010–2020) promulgated by Shanghai Municipality, the in-depth implementation of the Plan for Promoting the Development of Higher Education Cooperation in the Guangdong-Hong Kong-Macao Greater Bay Area promulgated by Guangdong Province, and the Reform and Development Plan of Higher Education in Zhejiang Province promulgated by Zhejiang Province (2000–2020) and other policy measures to promote synergy between education and the economy; However, the coupling coordination degree of Guizhou, Qinghai, Ningxia and Xinjiang provinces in the western region is at the bottom in each year, which is closely related to the decoupling of education and economic development in the western region.

Finally, the comparison between intermediate coordination and borderline dysregulation in the east and mild dysregulation and severe dysregulation in the west shows that there is a significant difference between the coordinated development of graduate education and high-quality economy in the east and west of China, and the development is unbalanced.

#### 4.1.3 Subregional analysis

(1) The change of two indices in four regions

In order to further compare the differences among different regions, this paper takes the eastern, central, northeastern and western regions divided according to economic development as an example, first takes the postgraduate education level of each year, the level of high-quality economic development and the mean value of the difference between the two and draws the following figure for analysis.

**Fig. 2:**
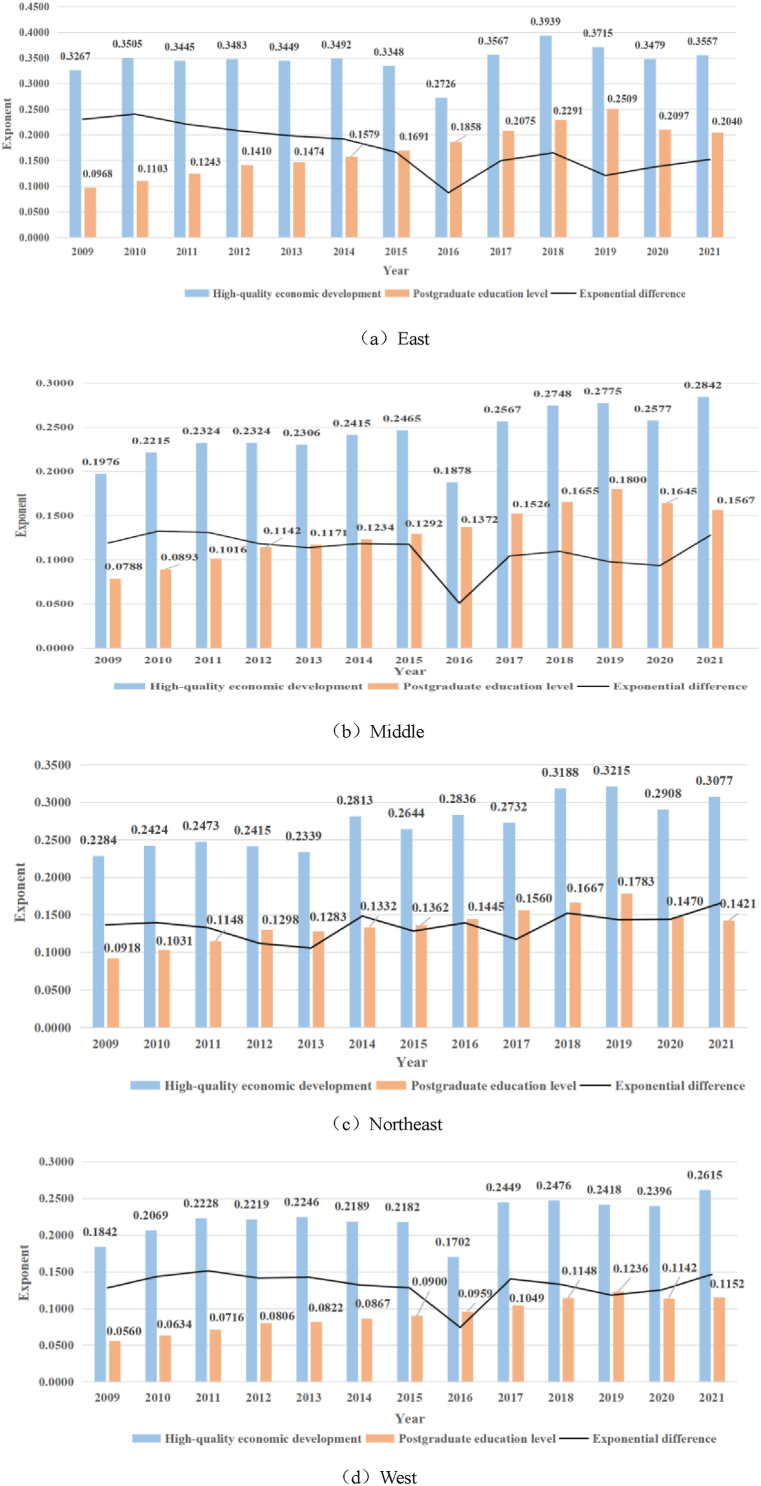
Comparison of graduate education and high-quality economic development in different regions.

In Fig. 2, figures (a), (b), (c) and (d) show the development trends and differences of high-quality graduate education and economic development in the eastern, central, northeastern and western regions, respectively. It can be found that the trend of high-quality graduate education and economic development in the eastern, central and western regions is similar to the national trend in Fig. 1 but the index value of the eastern region is higher than that of the central and western regions in each year, and the index value of the western region is the lowest in each year. Moreover, in 2016, due to the decline in the level of graduate education and the level of high-quality economic development, the result of a significant decline in both is not what we expected; The northeast region is slightly higher than the central region in value, and the postgraduate education level, the high-quality economic development level and the index difference of the two have shown a wave rise as a whole, which is related to the high concentration of colleges and universities in the northeast region and the high level of postgraduate education, but the economic development is relatively insufficient in the eastern region.

(2) The mean of coupling coordination degree of each region

**Fig. 3:**
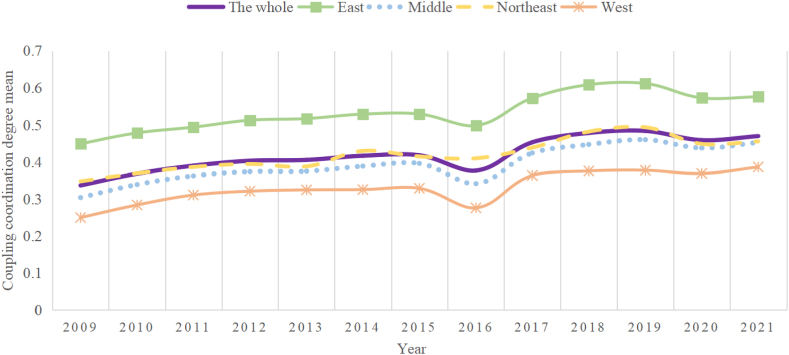
The mean of coupling coordination degree between graduate education and high-quality economic development.

In order to further analyze the differences in the coupling coordination degree of each region, this paper takes the average of the coupling coordination degree of the five regions according to each year, and the specific results are shown in Fig. 3 below.

In Fig. 3, it can be seen that the mean value of the coupled coordination degree in the five regions shows a slow upward trend as a whole, and continues to rise after the decline in 2016 and 2019. The mean value of the coupled coordination degree in the eastern region is much higher than the national scope, while the mean value in the central and western regions is lower than the national scope, and the mean value in the western region is lower. The mean of the coupling coordination degree in the central region fluctuates around the whole country, which is consistent with the conclusion that "there is a significant difference between the coordinated development of graduate education and high quality economy in China's eastern and western regions, and the development is unbalanced".

### 4.2 The dynamic evolution of the coupling coordination degree between graduate education and high-quality economic development

**Fig. 4:**
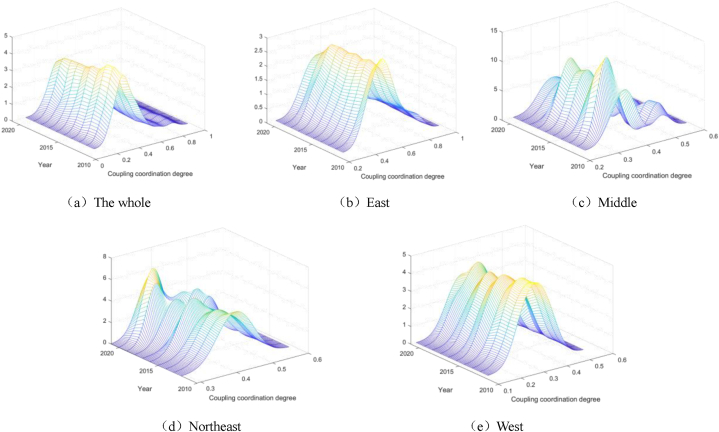
Kernel density dynamic coevolution estimation.

In order to further observe the variation of the coupling coordination degree between graduate education and high-quality economic development over time, based on the kernel density estimation method, this paper presents its dynamic evolution process from four perspectives: distribution location, distribution pattern of main peaks, distribution extensibility and number of peaks. Specific results are shown in Fig. 4 below.Which figure (a), (c), (b), (d) and (e), respectively, said the national, eastern, central, northeast and western regions high quality postgraduate education and economic growth in the coupling coordination degree of kernel density estimation.

#### 4.2.1 Distribution position

In the whole country, the dynamic synergistic effect between graduate education and high-quality economic development shows a left-skewed distribution mainly concentrated at about 0.5, which means that the coupling coordination degree is between the verge of misalignment and barely coordination, and the right-shifting phenomenon is not obvious. The distribution locations of the eastern region and the western region are similar to the national range, but the coupling coordination degree of the eastern region is concentrated between 0.5 and 0.6 (barely coordinated) and higher than the national level of 0.5, while the coupling coordination degree of the western region is concentrated between 0.4 and 0.5 (on the verge of imbalance). The reason is that the education and economic development of the eastern region is significantly more developed than that of the western region. The distribution position of the central region moved from left skew to right skew, and there was a significant inflection point in 2016, which may be due to the 13th Five-Year Plan for Promoting the Rise of the Central Region issued by the National Development and Reform Commission at the end of 2016, which greatly promoted the coordinated development of education and economy in the central region. In 2018, the northeastern region showed a significant right-skew, while the rest of the region was left-skew, and the peak value increased significantly, which may be due to the introduction of the Major Theoretical and Practical Issues of Northeast Education Serving the Comprehensive Revitalization and Development Strategy of Northeast China in 2018, which promoted the coordinated development of education and economy.

#### 4.2.2 Main peak distribution pattern

In the whole country, the coordinated development of graduate education and high economic quality showed a trend of first decreasing in height and then increasing in width, and then increasing after a significant decline in 2018. The distribution pattern of the main peaks in the eastern and western regions is similar to that of the whole country. In 2018, due to the reform of off-campus education and training institutions, the coordinated development of graduate education and economic quality in the eastern and western regions declined, and the decline rate in the eastern region was greater than that in the western region. The overall fluctuation and width of the main peak decreased in the central region. The height of the main peak in the northeast region increased but the width decreased, which means that the overall difference of coordinated development in these regions gradually decreased.

#### 4.2.3 Distributed ductility

The whole country, the central region and the northeast region all show a small degree of right-trailing phenomenon. Most regions have a low degree of synergistic effect, but there are also a small number of regions have a high degree of synergistic effect, which means that the coordinated development of graduate education and high quality economy is unbalanced. In addition, there is no obvious drag phenomenon between the eastern region and the western region. The education and economic development of the eastern region is quite high, resulting in a high synergistic effect on its overall development, while the western region is just the opposite.

#### 4.2.4 Crest number

The unimodal phenomenon exists in the whole country, the eastern region and the western region, and the coordinated development of graduate education and high economic quality is concentrated. In recent years, a large number of bimodal phenomena coexist in the central region, which means that the internal differentiation phenomenon is serious, and it is necessary to break the phenomenon of "middle depression" of coordinated development. In the northeast region, the single peak of low coupling coordination degree gradually evolved into the double peak of low and high coupling coordination degree co-existing with the development of time, and the differentiation is developing in the direction of promoting synergistic effect. The specific dynamic evolution characteristics are shown in Table 3 below.

**Table 3:** Dynamic evolution characteristics of synergistic effect between graduate education and high-quality economic development.

Region name	Distribution position	Main peak distribution pattern	Distributed ductility	Crest number
The whole	Left skewness	Height first down and then up, width becomes smaller	Right streaking	Unimodal
East	Left skewness	Height first down and then up, width becomes smaller	No	Unimodal
Middle	Right shift	The height decreases overall and the width becomes smaller	Right streaking	Doublet
Northeast	Move left and then right	Height goes up, width goes down	Right streaking	Unimodal or bimodal
West	Left skewness	Height first down and then up, width becomes smaller	No	Unimodal

### 4.3 Trend prediction of synergistic effect between graduate education and high-quality economic development

The long-term trend of the coupling coordination degree between graduate education and high-quality economic development is predicted according to the spatial Markov chain model. Firstly, the values to be predicted are discretized, and the values to be predicted are discretized into the lowest level (I), the lower level (II), the higher level (III) and the highest level (IV) by using the quartile method. The traditional Markov probability matrix without lag period is compared, and the specific results are shown in Table 4 below.

**Table 4:** Probability prediction matrix of coupling coordination degree between graduate education and high quality economic development.

Type	Type	I	II	III	IV	Sample size
No lag	I	0.7474	0.2316	0.0211	0.0000	95
	II	0.0989	0.7143	0.1868	0.0000	91
	III	0.0000	0.0795	0.8182	0.1023	88
	IV	0.0000	0.000	0.0233	0.9767	86
I	I	0.7500	0.2188	0.0313	0.0000	32
	II	0.0667	0.6667	0.2667	0.0000	15
	III	0.0000	0.0000	1.0000	0.0000	4
	IV	0.0000	0.0000	0.0000	1.0000	2
II	I	0.8378	0.1622	0.0000	0.0000	37
	II	0.1154	0.6154	0.2692	0.0000	26
	III	0.0000	0.1538	0.6923	0.1538	26
	IV	0.0000	0.0000	0.0000	1.0000	22
III	I	0.5000	0.4444	0.0556	0.0000	18
	II	0.0976	0.7805	0.1220	0.0000	41
	III	0.0000	0.0769	0.8462	0.0769	39
	IV	0.0000	0.0000	0.0000	1.0000	24
IV	I	0.0000	0.0000	0.0000	0.0000	0
	II	0.2000	0.6000	0.2000	0.0000	5
	III	0.0000	0.0000	0.8947	0.1053	19
	IV	0.0000	0.0000	0.0526	0.9474	38

In Table 4 above, it can be seen that if the lag period is not considered, first of all, the coupling coordination degree of graduate education and high-quality economic development has a certain probability of upward development, and the lowest level of coupling coordination degree has a 23.16 % probability of developing into a lower level of coupling coordination degree. There is 18.68 % probability that the lower level of coupling coordination degree will develop into the higher level of coupling coordination degree, and 10.23 % probability that the higher level of coupling coordination degree will develop into the highest level of coupling coordination degree. Secondly, with a certain uncertainty, the lower level, higher level and highest level of coupling coordination have a certain probability of developing to the lower level, for example, the coupling coordination at a higher level has a 7.95 % probability of developing to a lower level of coupling coordination, and the probability of developing to a higher level of coupling coordination at the highest level is 2.33 %. Finally, through the comparison between the two, it can be found that the probability of upward development of the coupling coordination degree is greater than the probability of downward development, which means that the synergistic effect between graduate education and high-quality economic development is developing in a good direction, but the higher the level of upward development, the lower the probability of occurrence.

After considering the lag period, the following conclusions are obtained. First, there is a "beggar-thy-neighbor" phenomenon, in the lowest level, lower level, higher level and highest level of coupling coordination degree, there is a great probability to maintain the development trend of the corresponding level, that is, the probability of the main diagonal is much greater than the probability of the non-main diagonal.It means that the industrial comprehensive water use efficiency of each city is to maintain a certain level of development; Second, there is a phenomenon of "neighborliness". The lower coupling coordination degree in type I, the lower and higher coupling coordination degree in type II, the lower and higher coupling coordination degree in type III and the lower coupling coordination degree in type IV have a trend of upward or downward development, and the probability of downward development is greater than that of upward development in type I with the lowest level. The strategic position placed on priority development is inseparable; Third, the higher the type associated with the coupling coordination degree, the probability of upward development first increases and then decreases. For example, the probability of the lower coupling coordination degree in type I progressing to one level is 26.67 %, the probability of the lower coupling coordination degree in type II progressing to one level is 26.92 %, and the probability of the lower coupling coordination degree in type III progressing to one level is 12.20 %. At the same time, the lower the type connected with the coupling coordination degree, the downward development first increases and then decreases. For example, the probability of the lower coupling coordination degree in type I developing to the next level is 6.67 %, the probability of the lower coupling coordination degree in type II developing to the next level is 11.54 %, and the probability of the lower coupling coordination degree in type III developing to the next level is 9.76 %. This also indirectly proves that the synergistic effect between graduate education and high-quality economic development in various provinces is affected by regional differences.

## 5 Discussion, conclusion and suggestion

### 5.1 Discussion

Graduate education promotes the high-quality development of social economy in the aspects of innovation, coordination, green, openness and sharing [[18], [19], [20], [21], [22]]. Meanwhile, the high-quality development of social economy determines the structure, scale, quality and efficiency of graduate education [[23], [24], [25], [26]], which complement each other and progress in synergy. However, with China's vast territory and abundant resources, the level of graduate education and high-quality economic development in different economic regions is different [34], and the synergistic development effect is also significantly different. The dynamic evolution prediction and analysis of the synergistic effect is conducive to speeding up the process of comprehensively building a modern socialist country.

However, this paper still has the following shortcomings, which can be further explored from the following three aspects in the next step: First, from the perspective of the research content, there is no detailed analysis of the reasons for the difference between graduate education and high-quality economic development, such as adopting the principal component analysis to judge, and laying the groundwork for substantive recommendations; Secondly, from the perspective of research methods, a deeper machine learning model can be adopted to improve the accuracy of dynamic evolution prediction. Finally, from the research conclusion, if city or county data can be used, the results of coupling coordination degree will be more accurate, and the suggestions can be more in-depth and practical.

### 5.2 Conclusion

Based on the panel data of 30 provinces (excluding Hong Kong, Macao and Taiwan) from 2009 to 2021, this paper measures the graduate education development index from four dimensions: scale, structure, quality and efficiency, and measures the economic high-quality development index from five dimensions: innovation, coordination, green, openness and sharing. At the same time, nuclear density estimation and spatial Markov chain model are used to predict the dynamic evolution of synergies in four economic regions, and the main conclusions are as follows:

First, from the measurement results, the economic quality development index of each region and each year is much higher than the graduate education development index. In most provinces of the country, graduate education and economic quality are relatively matched, but there are significant regional differences in the coordinated development of the two. The eastern region is higher than the central region, and the northeastern region is higher than the western region.

Second, from the perspective of dynamic evolution, the left-skew of coupling coordination degree is more significant, and the level of cooperative development is low, and only the northeast and central regions have a small amount of right-shift phenomenon. The width of nuclear density curves in the five regions decreased, and the overall difference of synergistic effects gradually decreased. In the whole country, the central region and the northeast region, there is a right tailing phenomenon, and the overall development of the synergistic effect is unbalanced. The unimodal phenomenon exists in both eastern and western regions, and the coupling coordination degree between graduate education and high-quality economic development is relatively concentrated, while the bimodal phenomenon in central and northeastern regions means that their internal differentiation is serious.

Third, from the results of trend prediction, there are coexistence of "beggar-thy-neighbor" and "neighborly kindness", and the higher the type connected with the coupling coordination degree, the probability of upward development increases first and then decreases. The lower the type connected with the coupling coordination degree, the downward development will first increase and then decrease, and it is difficult to achieve leap-forward development.

### 5.3 Suggestion

Although the synergistic effect between graduate education and high-quality economic development in various provinces in China shows an upward trend, it is still at a low level compared with European and American countries. Therefore, this paper puts forward the following three suggestions:

First, high-quality economic development provides a rich material foundation for graduate education. Economically developed cities tend to attract more high-level talents to settle and research, so it is necessary to improve the treatment of high-level talents in various provinces and cities, increase the investment in education, and solve the problems of settlement and transportation, so that high-level talents can devote themselves to the production and life of work.

Second, changes in the structure of postgraduate education should meet the needs of high-quality economic development. China's economic development has realized a rapid transformation to high-quality, in the international competition, social development is more urgent for high-level talents, so it is necessary to increase the education of master students, especially doctoral students, not only to increase enrollment efforts, but also to improve the quality of postgraduate education. Scientific research is not only based on the quantity and quality of papers, but also based on actual productivity.

Third, different reform measures should be implemented in different regions. The eastern region should continue to play a leading role and set an example for national education and economic development. The interaction between education and economy and other regions should be strengthened. The northeast region should strengthen economic development, turn the achievements of postgraduate education into capital export, retain the talents of colleges and universities, and implement the achievements of scientific research into the actual productive forces. The central and western regions should comprehensively strengthen postgraduate education and high-quality economic development, catch up with the eastern region, and introduce high-level talents from the East and exchange studies.

## Data availability statement

The datasets used and analyzed during the current study are available from the corre-sponding author on reasonable request.

## Funding statement

This research received funding from 2021 Research and Practice Project of Higher Education Teaching Reform in Henan Province, China: "Research and Practice on the Construction of Graduate Tutor Team for Professional degree in Business Administration (MBA)" (2021SJGLX072Y).

## CRediT authorship contribution statement

**Huang Zhiqi:** Writing – review & editing, Resources, Project administration, Methodology, Funding acquisition, Formal analysis, Data curation, Conceptualization. **Sun Fan:** Writing – review & editing, Writing – original draft, Visualization, Validation, Resources, Methodology, Formal analysis, Data curation, Conceptualization. **Zhou Yangmei:** Writing – review & editing, Formal analysis, Data curation. **Li Yan:** Writing – review & editing, Data curation, Formal analysis, Resources. **Li Zheng:** Validation, Writing – review & editing.

## Declaration of competing interest

The authors declare that they have no known competing financial interests or personal relationships that could have appeared to influence the work reported in this paper.

## Figures and Tables

**Table A1:** Evaluation system of graduate education and high-quality economic development indicators

Primary index	Secondary index	Three-level index	Stats	Specific meaning
High quality economic development index	Innovative development	GDP growth rate	+	Regional GDP growth rate
		R&d investment intensity	+	R&D expenditure/GDP of industrial enterprises above designated size
		Investment efficiency	–	Investment rate/GDP growth rate
		Technology trading activity	+	Technology trading turnover/GDP
	Coordinated development	Demand structure	+	Total retail sales of consumer goods/GDP
		Urban-rural structure	+	Urbanization rate
		Industrial structure	+	Output value/GDP of the tertiary industry
		Government debt burden	–	Government debt balance/GDP
	Green development	Elasticity coefficient of energy consumption	–	Energy consumption growth rate/GDP growth rate
		Wastewater per unit of output	–	Wastewater discharge/GDP
		Unit of exhaust gas produced	–	Sulfur dioxide emissions/GDP
	Open development	Dependence on foreign trade	+	Total imports and exports/GDP
		Proportion of foreign investment	+	Total foreign investment/GDP
		Marketization degree	+	Regional marketization index
	Shared development	Proportion of workers' compensation	+	Worker's compensation/GDP
		Elasticity of personal income growth	+	Per capita disposable income growth rate/GDP growth rate
		Urban-rural consumption gap	–	Per capita consumption expenditure of urban residents/per capita consumption expenditure of rural residents
		The proportion of public fiscal expenditure	+	The proportion of local expenditure on education, medical and health care, housing security, social security and employment in local budget expenditure
Graduate education development index	Scale	Enrollment scale	+	Graduate student enrollment index (Thousands of people
		Educational scale	+	Number of graduate students (Thousands of people)
	Structure	Doctoral ratio	+	Number of PHDS/Total number of graduate students
		International exchange	+	Number of Master's and doctoral students in China (persons)
	Quality	Fund input	+	Government expenditure on education (ten thousand yuan)
		Construction of teaching staff	+	Number of teachers undertaking national competitive scientific research projects (persons)
	benefit	Employment situation	+	Number of graduates in the employed population (persons)
		Educational contribution	+	Graduate Education Outstanding Achievement Award
